# Recent Advances in the Research and Application of Protective Composites for Stone Surfaces

**DOI:** 10.3390/ma19081545

**Published:** 2026-04-13

**Authors:** Qinghong Shi, Ting Zhao, Tao Yang, Lingmin Liao, Wei Dai, Xiaoyan Zhou, Xiaofeng Qin, Yun Dong, Wei Han

**Affiliations:** 1 Xiangjiaba Power Plant of China Yangtze Power Co., Ltd., Yibin 644612, China; 2 Changjiang River Scientific Research Institute, Changjiang Water Resources Commission, Wuhan 430010, China; dongyun@mail.crsri.cn (Y.D.); hanwei@mail.crsri.cn (W.H.); 3 Key Laboratory of Changjiang River of Ministry of Water Resources, Wuhan 430010, China

**Keywords:** stone surface protection, composite materials, nanocomposites, hydrophobic coatings, durability

## Abstract

The degradation of stone surfaces resulting from natural aging and environmental factors poses significant challenges to material durability and esthetics. This review systematically summarizes recent advances in composite protective materials designed to enhance stone surface preservation. It focuses on the classifications and fundamental properties of organic polymer-based materials, inorganic nanocomposites, and multifunctional protective coatings, emphasizing their physicochemical, mechanical, and environmental performance. The review further analyzes case studies across various stone substrates, such as marble, limestone, sandstone, and granite, highlighting substrate-specific coating compatibility and protective effectiveness against water ingress, biological colonization, graffiti, and pollutant deposition. Critical factors influencing protective performance, including stone mineralogy, coating composition, environmental conditions, and application protocols, are elucidated. Finally, existing challenges and future research directions are identified, underscoring the need for environmentally friendly, multifunctional, and durable composite coatings with enhanced substrate adaptability and simplified application processes.

## 1. Introduction

Stone surfaces are susceptible to degradation due to a combination of natural aging and adverse environmental conditions, leading to various forms of surface damage and loss of structural integrity [1,2,3]. The deterioration process involves complex interactions between intrinsic stone properties (such as mineralogy, texture, and porosity) and external factors, including weather, atmospheric pollution, temperature fluctuations, biological agents, and water penetration [4]. Water, in particular, is identified as a primary driver of degradation, facilitating processes such as salt crystallization, freeze–thaw cycles, acid rain corrosion, and microbial colonization, which induce mechanical stress, flaking, and material loss [5]. For instance, salt crystallization is a significant deterioration mechanism, leading to mechanical stress and eventual loss of material integrity, while water infiltration can cause internal moisture accumulation and salt crystallization stress of up to 30 MPa in porous stones like sandstone [6]. Biological colonization, including biofilm formation, further accelerates degradation by promoting chemical reactions and physical weathering [7,8,9]. In addition, although no single universally adopted standard explicitly mandates protective treatment solely for stone surfaces exposed to high temperature variations, several natural stone standards provide an important technical basis for evaluating such risks and for selecting appropriate protective strategies. For example, standards concerning thermal shock resistance, linear thermal expansion, and freeze–thaw resistancecan be used to assess the vulnerability of stone materials under severe thermal fluctuations and to support performance-based protection design [10,11].

Traditional stone protection systems mainly include organic polymeric materials and inorganic consolidating components [12,13,14,15,16]. Organic polymeric materials, such as acrylic-based products, have been widely used, but they may suffer from aging, yellowing, moisture trapping, and insufficient long-term durability. Inorganic consolidating systems have also been extensively investigated; among them, calcium hydroxide-based systems have attracted considerable attention because of their chemical compatibility with limestone-like substrates such as marble and travertine. In such systems, the protective and consolidating effect is closely associated with the carbonation-related transformation of Ca(OH)_2_ [17]. However, inorganic consolidants often still require multiple applications and may show limited penetration depth, substrate incompatibility, and environmental instability under certain conditions. These limitations have motivated the development of composite protective materials, which combine the synergistic properties of organic and inorganic components to address multiple degradation mechanisms simultaneously.

Composite materials, such as inorganic phosphate/polymer hybrids [18,19,20,21], organic–inorganic nanocomposites [17,22,23,24], and nanoparticle-reinforced coatings [6,25,26,27,28,29], offer enhanced performance by integrating beneficial properties like consolidation, hydrophobicity, self-cleaning [30,31,32], antimicrobial activity [33,34], and UV resistance [35]. For example, hybrid coatings based on silane/siloxane matrices with nanoparticles (NPs) (e.g., TiO_2_, SiO_2_, ZnO) have demonstrated improved mechanical resistance, water repellency, and durability. Additionally, biocompatible composites, such as chitosan/hydroxyapatite systems, provide environmentally friendly solutions with good compatibility and resistance to salt-induced deterioration. The design of such composites aims to balance functionality (e.g., water vapor permeability and esthetic preservation) with durability, ensuring long-term protection without compromising the stone’s historical and artistic value [36,37,38].

## 2. Classification of Stone Surface Protective Materials

Stone surface protective materials are discussed primarily according to the dominant function of the protection system rather than by strict chemical composition alone. Given the increasing overlap and hybridization among current protective systems, a purely composition-based classification is often insufficient to reflect their practical roles and protective mechanisms. Accordingly, the following discussion considers stone protective materials from two complementary perspectives, namely material composition and dominant protective function, to better present their design features and functional characteristics.

### 2.1. Organic Polymer-Based Protective Materials

Organic polymer-based protective materials, including acrylics and biodegradable polymers, play a crucial role in stone surface protection, often formulated as hydrophobic coatings or organic–inorganic nanocomposites to enhance performance [39].

**Figure 1:**
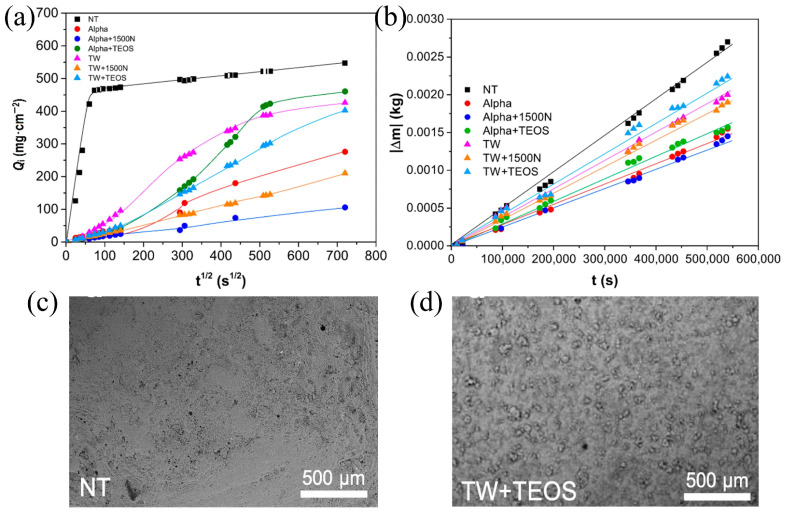
Moisture transport behavior and surface morphology of Vicenza stone before and after treatment: (**a**) capillary water absorption curves and (**b**) water vapor permeability of untreated and coated specimens; SEM images of (**c**) the untreated surface and (**d**) the surface treated with the TW+TEOS formulation [42].

Acrylic polymers are widely used, with types ranging from basic acrylics and vinyl acetate to modified variants such as fluorinated acrylics and water-borne systems [39]. Commercial acrylic resins like Paraloid B72 are common, though they suffer from poor resistance to thermal and photooxidation aging [7,40]. To address these limitations, acrylics can be modified with inorganic NPs (e.g., Ca(OH)_2_, TiO_2_, SiO_2_) to enhance conservation performance, and water-borne formulations such as photo-polymerizable methacrylate nanocomposites offer environmentally friendly options with improved durability. Fluorinated acrylic polymers, incorporating monomers like 1H,1H,2H,2H-perfluorodecyl methacrylate (XFDM) and 1,1,1,3,3,3-hexafluoroisopropyl methacrylate (HFIM), exhibit enhanced water repellency (static contact angles up to ~108°) and photostability due to strong C–F bonds, outperforming non-fluorinated counterparts like Paraloid B72 in resisting photooxidative degradation [41]. For instance, acrylic resin solutions (e.g., Twinswet) modified with additives like tetraethyl orthosilicate (TEOS) or amino-functional polysiloxane (TegoPhobe 1500 N) show increased hydrophobicity (water contact angles (WCAs) ~138–140°) and reduced capillary water absorption (Figure 1,b), with TEOS promoting in situ silica nanoparticle formation to enhance surface roughness (Figure 1c,d) [42].

In 2011, Khallaf et al. [43] investigated the use of an acrylic polymer as a protective coating on sandstone and limestone, focusing on its adsorption mechanism and effects on physical and mechanical properties. Fourier-transform infrared spectroscopy (FTIR) and electrokinetic measurements demonstrated that polymer adsorption on stone surfaces is mainly physical, with no chemical reaction altering the surface potential, and that the polymer forms a protective film on the stone surface. After treatment, significant improvements were observed in physical properties, such as increased bulk density (from 1.9 to 2.3 g/cm^3^), reduced porosity (from 15.8% to 2.7%), and decreased water absorption, along with notably increased mechanical strength, including compressive strength exceeding 20 MPa. Artificial aging tests (wet–dry cycles, salt crystallization, and acid water exposure) confirmed the durability of the polymer coating, though some coating cracks appeared after aging. The polymer coating acts as a water-repellent layer that reduces water ingress and contamination, thereby increasing stone durability and preserving surface integrity.

Biodegradable polymers, such as zein, chitosan, polyhydroxybutyrate (PHB), poly-L-lactide (PLA), and poly(hydroxyalkanoate)s (PHAs), offer environmentally friendly and reversible protection. PHB and high-molecular-weight PLA (HMWPLA) increase marble surface hydrophobicity (WCA 86° for PHB, 99° for HMWPLA) and reduce SO_2_-induced gypsum formation (HMWPLA achieving a 60% reduction) [44]. PHAs, including PHB and its copolymer PHBVV, provide “reversibility by biodegradation,” naturally degrading after losing water repellency to avoid permanent residues. They exhibit comparable water repellency to commercial silane/siloxane treatments, with PHBVV generally outperforming PHB, and maintain good water vapor permeability and esthetic compatibility (ΔE* < perceptible thresholds) [45]. Nanocomposite biodegradable coatings, such as PLA/montmorillonite (MMT) with 5 wt% MMT, show enhanced hydrophobicity (WCA 108°), reduced capillary water absorption (66% reduction), and inhibited gypsum formation under acidic conditions, attributed to exfoliated nanoclay structures creating impermeable barriers [46].

### 2.2. Inorganic Nanocomposite Materials

Inorganic nanocomposite materials for stone surface protection encompass a diverse range of compositions, including metal oxides (e.g., TiO_2_, ZnO, SiO_2_), calcium oxalate, hydroxyapatite, and their hybrids with polymers, offering enhanced protective functionalities through nano-scale effects.

In 2015, Munafò reported that TiO_2_ NPs (mean diameters 5–50 nm) exhibit distinct properties such as near transparency in the visible spectrum and strong UV absorption, differing from microscaled TiO_2_. These NPs possess photoinduced features activated by UV light, including photocatalysis (generation of reactive charge carriers for oxidizing organic compounds) and superhydrophilicity (high wettability creating uniform water films), which synergize to produce self-cleaning effects on treated stone surfaces [47]. In 2024, Ilies applied nano-TiO_2_ (10–30 nm, rutile form) as protective coatings on fossiliferous limestone, forming a homogeneous layer that fills micro-cracks and pores, enhancing compressive strength (14.57 MPa for 2% and 19.59 MPa for 5% coatings vs. 9.5 MPa for untreated samples) and maintaining coverage integrity after six months of simulated extreme conditions [48]. Additionally, in 2022, Torabi-Kaveh found that dispersing TiO_2_ polymorph NPs (anatase: 10–25 nm; rutile: 50 nm) in orthophthalic polyester resin significantly increased the durability of travertine stone surfaces during artificial aging, with resin–anatase TiO_2_ exhibiting the best performance [49]. TiO_2_ has also been integrated into hybrid systems; in 2013, Kapridaki synthesized a transparent hydrophobic SiO_2_-TiO_2_-PDMS nanocomposite via sol–gel, where TiO_2_ contributed to photocatalytic self-cleaning while maintaining hydrophobicity and water vapor permeability [50].

**Figure 2:**
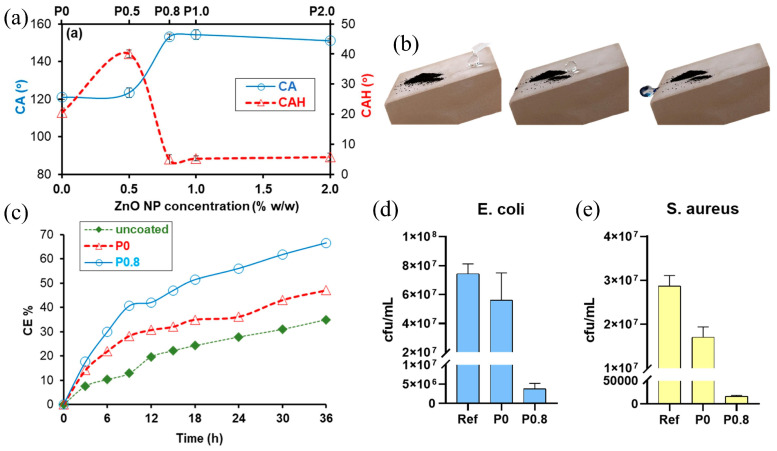
Multifunctional performance of ZnO-containing coatings on limestone: (**a**) contact angle (CA) and contact angle hysteresis (CAH) of water drops on coated limestone samples vs. ZnO NP concentration; (**b**) image sequence illustrating the lotus effect self-cleaning behavior of a contaminated surface treated with the P0.8 coating; (**c**) photocatalytic cleaning efficiency toward methylene blue under UV irradiation; and antibacterial activity against (**d**) *E. coli* and (**e**) *S. aureus* relative to the reference condition [51].

ZnO nanocomposites have also shown promise. In 2023, Manoudis developed a multifunctional coating by dispersing ZnO NPs (<100 nm) into an aqueous silane-based system, achieving superhydrophobicity due to hierarchically rough surface morphology, along with photocatalytic self-cleaning and biocidal activity [51]. By incorporating 0.8% (*w*/*w*) ZnO nanoparticles, the coating achieves a highly non-wetting state characterized by a WCA of 153° and a small contact angle hysteresis (CAH) of about 5° (Figure 2a). This superhydrophobicity created a physical self-cleaning mechanism known as the lotus effect, where rolling water drops effectively carry away dirt and surface contaminants (Figure 2b). In addition to this physical mechanism, the ZnO nanoparticles demonstrate strong photocatalytic chemical self-cleaning capabilities under UV-A radiation, which successfully degrades organic pollutants like methylene blue stains from the stone (Figure 2c). Furthermore, the composite coating exhibits remarkable biocidal activity by significantly hindering the incubation and growth of harmful microorganisms. Even at the low optimal concentration of 0.8%, the nanoparticles provide powerful antibacterial protection, with inhibition rates reaching 94.8% against *E. coli* and 99.9% against *S. aureus* (Figure 2d,e).

**Figure 3:**
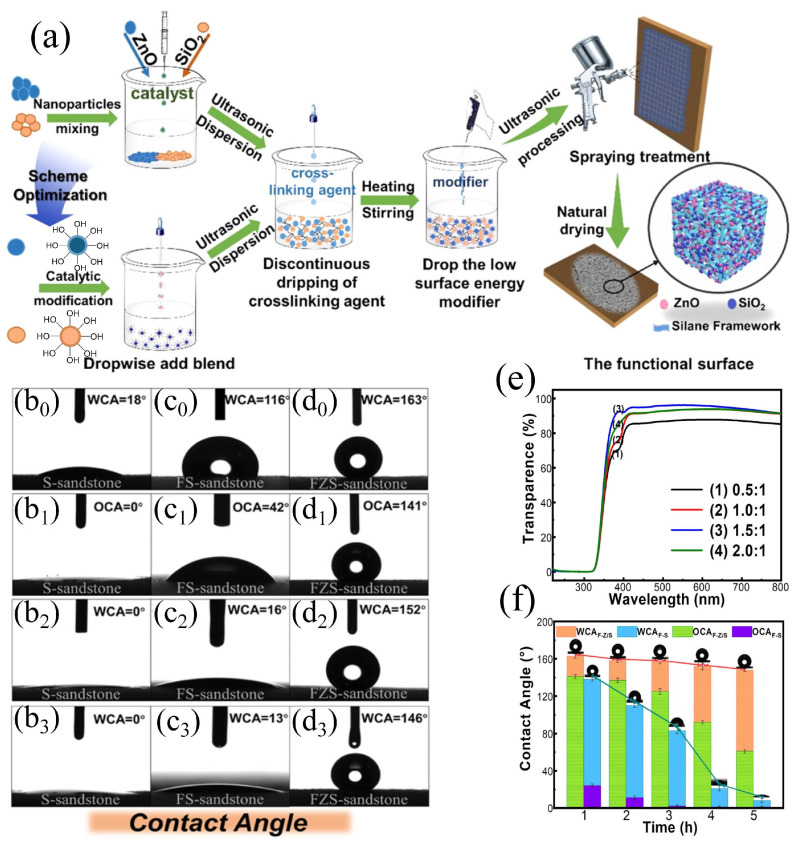
Fabrication and protective performance of the ZnO/SiO_2_-based coating for sandstone: (**a**) schematic illustration of the preparation process; (**b_0_**–**d_3_**) comparison of the wetting behavior of sandstone surfaces treated with SiO_2_, fluorinated ZnO, and FZS hybrid coatings under the investigated conditions, including the initial WCA and OCA of the coating (x_0_), (x_1_) on sandstone, and the WCA of the sandstone surface after temperature–humidity aging and natural aging (x_2_), (x_3_); (**e**) UV–visible transmittance spectra of coatings with different ZnO/SiO_2_ ratios; and (**f**) water and oil repellency retained after 300 h of UV exposure [52].

In 2022, Chai prepared a ZnO-SiO_2_ nanocomposite (FZS) cross-linked with silane and modified by fluorocarbon (Figure 3a), exhibiting excellent amphiphobicity and remarkable UV resistance (Figure 3e,f) [52]. The sprayed material exhibited outstanding amphiphobicity, achieving WCA > 160° and OCA > 140° (Figure 3(b_0_–d_3_)). Furthermore, it provided exceptional UV resistance, with an absorption rate approaching 100%. Extensive aging tests demonstrated that the coating maintained its superior hydrophobic characteristics almost perfectly even after 300 h of continuous UV irradiation (Figure 3e,f).

Calcium oxalate-based nanocomposites offer enhanced compatibility with carbonate stones. In 2013, Maravelaki synthesized a hydrophobic nanocomposite by incorporating nano-calcium oxalate (CaOx) into a TEOS-derived silica matrix with PDMS via sol–gel [36]. Oxalic acid catalyzed CaOx formation and TEOS hydrolysis, resulting in a hybrid with particle sizes of 7–700 nm, strong interfacial compatibility with limestone, and improved mechanical properties. The PDMS component imparted hydrophobicity (WCA > 90°) and reduced water capillary absorption by ~94.4%, with minimal color change. Kapridaki et al. [50] also noted that oxalic acid contributes to CaOx formation on stone surfaces, which is more stable and weather-resistant than calcite.

Hydroxyapatite (HAp) nanocomposites, combined with organic polymers, provide unique protective features. In 2024, Hafez developed a nanostructured hybrid coating of HAp nanocrystals with polyelectrolyte multilayers (polyethyleneimine (PEI) and polyacrylic acid (PAA)) via spray layer-by-layer on marble [37]. The HAp nanocrystals, synthesized via an aqueous route with PEI, formed a trabecular bone-like structure with chemical affinity, weathering resistance, and self-healing properties, demonstrating enhanced multifunctional protection through controlled surface functionalization.

### 2.3. Functional Protective Coatings

Functional protective coatings are essential for stone surface preservation by providing enhanced functionalities, with hydrophobicity being a key focus to mitigate water-induced deterioration.

**Figure 4:**
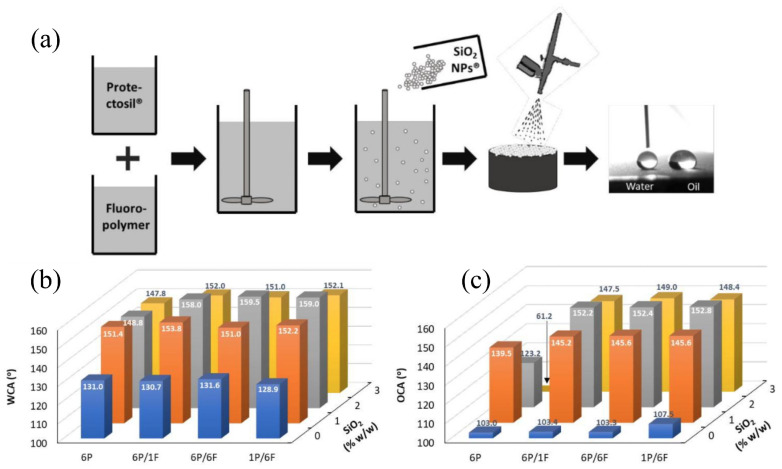
Preparation route and wetting performance of the superamphiphobic coating system on marble: (**a**) schematic illustration of the fabrication process; (**b**) water contact angles and (**c**) oil contact angles of coatings prepared with different P (Protectosil)/F (Fluoropolymer) ratios and SiO_2_NP contents [56].

Silanes and siloxanes are valued for their chemical stability and hydrophobic properties, with alkoxysilanes (e.g., tetraethyl orthosilicate, TEOS) and siloxane-based polymers being prominent examples. Alkoxysilanes penetrate stone pores via low viscosity, forming a three-dimensional silica network through hydrolysis (alkoxy groups to silanol) and polycondensation, with chemical bonding to stone surfaces via Si–O–Si bonds. Siloxane polymers orient hydrophilic silanol groups inside pores and hydrophobic alkyl chains outward, creating a water-repellent yet vapor-permeable layer. However, alkoxysilanes on carbonate stones may suffer from gel cracking and poor compatibility; modifications include adding drying control additives (e.g., formamide), surfactants (e.g., n-octylamine), NPs (SiO_2_, TiO_2_), or elastic segments (e.g., hydroxyl-terminated polydimethylsiloxane) to improve flexibility and reduce cracking [53]. Advanced formulations, such as UV-vis light-curable hybrid methacrylic–siloxane resins modified with organic-coated TiO_2_ nanorods, enable rapid in situ photopolymerization, forming uniform, hydrophobic coatings with photocatalytic self-cleaning properties [54,55]. Blending silanes (e.g., Protectosil SC Concentrate) with fluoropolymers and silica NPs (Figure 4a) can achieve superamphiphobicity (WCA and oil contact angles (OCAs) > 150°) via combined surface chemistry and roughness modification, with good durability under harsh conditions (Figure 4b,c) [56]. Siloxane-based oligomeric resins (e.g., Alpha^®^ SI30) modified with TEOS, also exhibit high hydrophobicity and breathability, outperforming some acrylic resins in durability.

SiO_2_ nanocomposites, often hybridized with polymers, are another important category. In 2012, Xu prepared a TEOS-based hybrid containing flexible hydroxyl-terminated polydimethylsiloxane (PDMS-OH) and colloidal silica particles using n-octylamine as a catalyst [57]. The colloidal silica, synthesized by TEOS hydrolysis in ethanol with ammonium hydroxide, when incorporated into the TEOS/PDMS matrix, reduced gel cracking, increased pore size, and enhanced hydrophobicity (maximum WCA 123° at 0.2% silica concentration), with acid rain resistance comparable to commercial fluoro-organosilanes. In 2008, Manoudis modified a commercial siloxane with 7 nm hydrophilic silica NPs, achieving superhydrophobicity (WCA 160°) on marble via micro- and nano-scale roughness, with low contact angle hysteresis confirming high water repellency [58].

**Figure 5:**
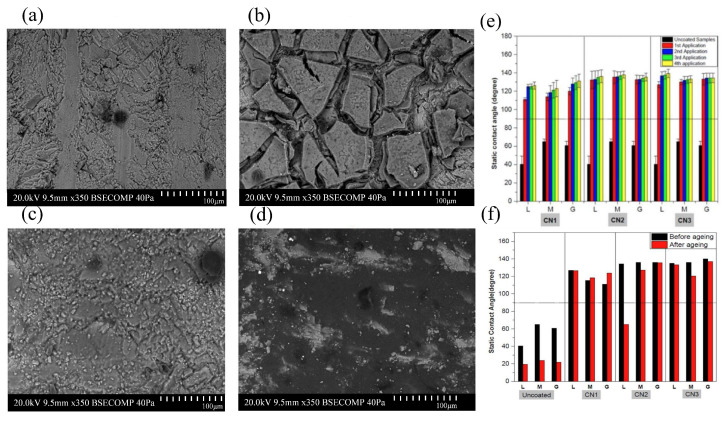
Surface morphology and hydrophobic response of marble before and after treatment with commercial products: SEM images (350×) of (**a**) the untreated surface and surfaces receiving four applications of (**b**) CN1, (**c**) CN2, and (**d**) CN3; (**e**) WCA of different lithotypes during optimization of the number of coating applications; and (**f**) WCA measured before and after accelerated aging for specimens coated at 4 °C while the stone substrates were maintained at room temperature [59].

Hashim studied the effect of coating and stone substrate temperatures, as well as the number of coating applications, on the effectiveness, compatibility, and durability of commercial hydrophobic coatings to prevent water penetration and subsequent stone alteration [59]. The results revealed that while more than one application increases coating hydrophobic effectiveness, it frequently leads to changes in the esthetic appearance of natural stone, including whitening and darkening of the substrate’s original hues, and additional applications beyond the second yield diminishing returns and may induce coating defects or cracks. Static contact angle measurement results demonstrated that all three commercial coatings (CN1: potassium methyl siliconate; CN2: fluorocarbon polymer; CN3: silane/siloxane with modified fluorinated additives) impart water-repellent properties, with CN2 showing the highest static contact angles indicative of superior hydrophobicity, followed by CN3. SEM and SEM-EDS analyses revealed that multiple applications of CN1 formed a thick, non-uniform coating with cracks, while CN2 and CN3 produced smoother, more uniform coatings. Accelerated aging tests indicated that CN1 and CN3 coatings, which penetrate and adhere strongly within substrate pores, maintain hydrophobicity better over time compared to CN2, which loses effectiveness especially on porous limestone (Figure 5a–e). Water vapor permeability assessments showed that CN3 reduces permeability in more porous limestone but maintains it in less porous marble and granitoid, evidencing compatibility differences related to stone microstructure. The combination of hydrophobic silane/siloxane penetration and fluorinated compound surface repellency in CN3 contributes to durable, effective water-repellent performance without severely compromising breathability. The study also identified optimal application parameters, showing that applying the hydrophobic coatings at 4 °C onto natural stone substrates at room temperature maximizes hydrophobic effectiveness while maintaining practical feasibility. The commercial coating composed of silane/siloxane with modified fluorinated additives (CN3) was found to be the most effective and durable hydrophobic solution (Figure 5f).

## 3. Performance Evaluation and Environmental Adaptability of Stone Protective Materials

### 3.1. Physicochemical Performance Assessment

Physicochemical performance assessment of stone protective coatings involves a combination of methods to evaluate key properties such as water repellency, capillary absorption, vapor permeability, color change, and chemical stability [50]. Water repellency is primarily evaluated through static WCA measurements, with superhydrophobicity typically defined as WCA > 150° [38,60]. In addition to static WCA, dynamic wettability parameters, including CAH, sliding angle (SA), and roll-off angle, are critical for assessing practical water repellency, as coatings with low CAH (<10°) and low SA ensure effective water shedding [61]. Advanced techniques, such as using custom-made automated devices (e.g., Kerberos) to monitor liquid interface deformation, spreading, and sliding during sample tilting, provide quantitative insights into dynamic wetting behavior [56].

Capillary water absorption, a key indicator of liquid water ingress resistance, is commonly assessed via gravimetric sorption techniques, measuring water uptake per unit area over time. Performance is quantified by parameters such as reduction in water uptake (e.g., ~79.8% for superhydrophobic composite coatings [6]) or water absorption inhibition efficiency (WIE), with values exceeding 92% indicating excellent repellence. For calcareous stones, absorption coefficients (ACs) and relative capillarity indices (RCIs) are also used to evaluate the effectiveness of hydrophobic protection, both before and after aging.

Water vapor permeability, essential for maintaining stone breathability, is also evaluated. Performance is typically reported as the relative decrease in vapor permeability compared to untreated substrates, with acceptable coatings generally showing a relative decrease below 20% and retaining over 80% of the original vapor permeability, ensuring moisture vapor transmission to prevent trapped moisture damage.

Color change assessment is conducted using the CIE Lab* colorimetric system, measuring total color difference (Δ*E*) between treated and untreated surfaces. Minimal Δ*E* values are critical for preserving esthetic appearance, with thresholds such as Δ*E* < 2, ΔE* ≈ 2.93 [6], or ΔE* < 3 [62] considered acceptable, as these are below perceptibility limits. Higher ΔE* values (e.g., <5) may still be tolerated depending on application requirements [63].

Chemical stability is evaluated through exposure to harsh environments, including acidic (pH = 1 to 5) and alkaline (pH = 13) solutions, saltwater immersion, and accelerated aging tests. For instance, acid rain simulation (pH = 5.0) and saltwater (1.0 wt% Na_2_SO_4_) immersion tests demonstrate coating resistance to environmental corrosion, with retained high hydrophobicity (WCA ~153° after cycles [6]). Chemical changes upon aging are analyzed via FTIR, which tracks degradation markers such as carbonyl band intensity, to assess photooxidative stability. Additionally, resistance to UV radiation, thermal cycling, and mechanical abrasion is integrated into comprehensive stability assessments, ensuring coatings maintain performance under long-term environmental exposure.

### 3.2. Mechanical Properties and Durability Testing

Mechanical properties of stone can be significantly influenced by protective materials, with both enhancing and deteriorating effects observed. For instance, in 2024, Li developed a flexible–rigid hybrid siloxane oligomer consolidant that improves the mechanical properties of weathered stone, with treated samples achieving a Leeb hardness of approximately 410 HL and compressive strength of 8.98 MPa [64]. This improvement is attributed to the formation of a continuous three-dimensional network inside the stone, combining flexible linear Si-O-Si segments from di-alkoxysilane with rigid three-dimensional silicon–oxygen domains from tetra-alkoxysilane, which mitigates volume shrinkage and cracking during curing and drying. Conversely, adverse mechanical effects have been reported for hydrophobic coatings on porous limestones: in 2024, Lisci found that after freeze–thaw cycles, treated porous limestones exhibited a significant decrease in flexural strength (up to −52%) and larger reductions in longitudinal pulse velocity and elastic modulus, indicating structural damage due to altered water migration and mechanical behavior differences between the coating layer and the stone matrix [65]. Adhesion, a critical mechanical property, was evaluated by Hafez via adhesion pull-off tests on marble samples coated with polyelectrolyte-hydroxyapatite multilayer (PHM) and HAp/PEI coatings, demonstrating good bonding efficacy without surface preparation [37].

Durability testing under various environmental stressors has been conducted to assess protective materials. For thermal shock resistance, in 2022, Torabi-Kaveh evaluated three coating products, including industrial resin, resin–anatase NPs, and resin–rutile NPs on travertine, conducting 40 cycles of accelerated aging tests based on thermal shock at 70 °C [49]. They found that resin-TiO_2_ nanoparticle hybrid coatings significantly increased surface durability, with the anatase nanoparticle product showing the best performance, including minimal mass changes. Similarly, in 2024, Lisci reported that thermal shock causes thermal stress damage due to mineral expansion/contraction differences, though it results in less mechanical degradation difference between treated and untreated stones compared to freeze–thaw cycles [65].

Salt crystallization testing by Torabi-Kaveh revealed that salt crystallization induces microcracking (especially in high-porosity stones), and resin-TiO_2_ hybrid coatings mitigated such damage related to salt crystallization pressures, as evidenced by weight loss, water absorption index, and surface hardness measurements [49].

For freeze–thaw cycles, in 2024, Lisci demonstrated that hydrophobic coatings cause treated stones to retain more water due to partial pore saturation, impeding natural water release during thawing and generating internal crystallization pressure [65]. This leads to premature mechanical damage, increased open porosity, and microcracking, whereas untreated porous stones maintain structural cohesion better by allowing natural water absorption and release.

Acid resistance was evaluated by Hafez’s team using scratch-dissolution experiments: marble samples coated with a nanostructured hydrophilic self-healing hybrid coating were scratched at different penetration levels and exposed to an acidic aqueous solution (pH 5.0) for three days, showing resistance to acid attack and less weathered surfaces [37].

Regarding dynamic load-related mechanical response, the role of protective materials is generally indirect rather than purely energy-dissipative. Penetrating consolidants or flexible–rigid hybrid systems can improve internal cohesion, interfacial bonding, and stress transfer within weathered stone, thereby helping to retard crack propagation and mitigate mechanical deterioration under cyclic actions. For example, flexible–rigid hybrid siloxane consolidants were reported to form a continuous three-dimensional reinforcement network inside weathered stone, leading to improved hardness and compressive strength. In contrast, some hydrophobic surface coatings, particularly on porous limestones, may negatively affect mechanical behavior under freeze–thaw conditions by hindering natural water release and creating mechanical incompatibility between the coating layer and the stone substrate, which can intensify internal pressure, microcracking, and the loss of flexural strength and elastic modulus. In addition, good adhesion is an important prerequisite for favorable mechanical performance, because strong interfacial bonding can facilitate load transfer and reduce stress concentration at the coating–stone interface. Therefore, the influence of protection on dynamic load-related response is substrate- and material-dependent and should not be regarded as universally crucial or negligible.

### 3.3. Environmental Compatibility and Substrate Interaction

The environmental adaptability and compatibility of stone protective coatings with substrates involve complex interactions encompassing chemical, physical, and esthetic aspects, as well as long-term stability, reversibility, and impacts on microstructure and porosity. The interaction between binder, aggregates, and stone substrate determines compatibility, with critical parameters including water transfer properties (vapor permeability), modulus of elasticity, thermal expansion behavior, adhesion, visual appearance, and porosity [47]. For instance, repair mortars should ideally match the stone’s porosity and vapor transmission to avoid water retention, while mismatched thermal expansion (e.g., dense Portland cement mortars with high thermal expansion coefficients) can cause stone damage under thermal cycles. Incompatibility cases, such as lime mortars inducing granite damage via calcium ion migration and gypsum formation, highlight the importance of chemical compatibility. Additionally, visual appearance must balance esthetic harmony with functional properties, as changes to meet visual demands can affect other critical attributes.

Biological factors also influence environmental adaptability. Pozo-Antonio noted that biological colonization (e.g., fungi, lichens) interacts physically and chemically with granite substrates, causing mineralogical transformations, physical cracking via hyphal penetration and humidity-driven expansion/contraction, and esthetic degradation through dark pigmentation, which obscures the stone’s artistic value [66].

## 4. Application Cases and Effectiveness Analysis

### 4.1. Protective Applications on Various Stone Types

**Figure 6:**
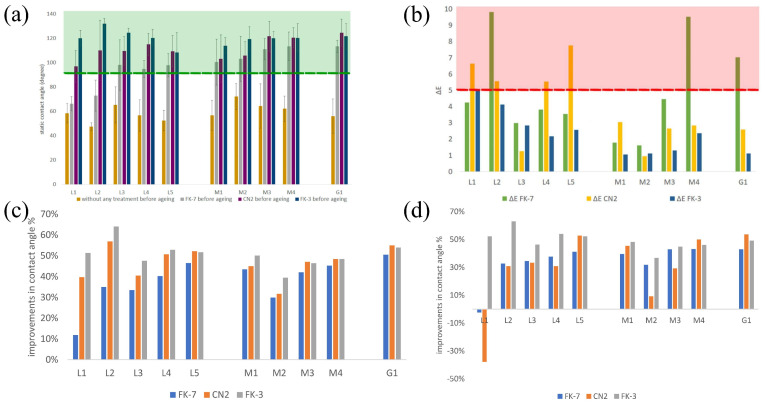
Comparative evaluation of commercial hydrophobic treatments on different stone substrates: (**a**) initial water-repellent performance, expressed by the WCA of untreated and coated mock-ups before aging; (**b**) color variation (ΔE*) induced by FK-7, CN2, and FK-3; (**c**) relative hydrophobic improvement produced by each coating; and (**d**) retention of hydrophobic enhancement after artificial aging relative to the untreated condition [68].

Protective coatings have been extensively studied and applied on various stone types, with case studies demonstrating tailored performance based on stone mineralogy and properties. For marble, in 2024, Hafez et al. developed a nanostructured hydrophilic and self-healing hybrid coating based on HAp nanocrystals combined with polyelectrolyte PEM, applied in situ on Greek marble (dolomitic medium-grained and calcitic fine-grained) via spray layer-by-layer surface functionalization [37]. The PHM coatings exhibited chemical affinity, esthetic compatibility, weathering resistance, and reversibility, with adhesion pull-off tests, acid dissolution experiments, and water uptake analyses validating strong bonding, acid resistance, and reduced water absorption. Naidu et al. [8] compared acid resistance of HAP, calcium oxalate, and calcium tartrate coatings on Carrara marble, finding that HAP coatings generated from 1M diammonium hydrogen phosphate with calcium chloride addition reduced the calcite dissolution rate by approximately 40% by improving coating coherency and substrate coverage. Sbardella et al. [62] applied water-borne acrylate-based hybrid coatings containing silica NPs on Carrara marble, where 5 wbm% silica content enhanced hydrophobicity (WCA from ~55° to above 80°), reduced water absorption, and improved photostability, with smaller colorimetric changes after accelerated aging. Gkrava et al. dispersed hydrophobic calcium carbonate NPs modified with dodecanoic acid in KSE100 silicic acid ester solution, spraying them on marble specimens to form superhydrophobic coatings with negligible color impact and good durability against rain, freeze–thaw cycles, and UV radiation [67]. Shu et al. evaluated a TiO_2_-modified sol coating material on marble, which increased acid resistance by 1.75 times compared to traditional coatings and maintained water vapor permeability [63]. Armal et al. included four marble types in case studies testing commercial hydrophobic coatings (FK-7, CN2, FK-3), with FAKOLITH FK-3 Plus Nano (modified C6 fluorinated compounds) showing the highest effectiveness for carbonate stones [68]. Results demonstrated that FK-3 protected porous materials by producing static WCA > 90°across all limestone and marble specimens (Figure 6a). The application maintained acceptable color stability (Figure 6b) and preserved adequate water vapor permeability. After artificial weathering simulations, the surfaces exhibited remarkable durability and retained their water-repellent properties (Figure 6c,d). Dias et al. tested commercial coatings on two marbles (Rosa JPL and Golden Brown), finding modified silanes and siloxanes derivatives most compatible and effective [69].

In addition, sulfate ions are particularly important for carbonate stones because they can promote gypsum formation and crystallization-related stresses [70], while chloride ions are highly mobile and hygroscopic, facilitating repeated dissolution–crystallization cycles and moisture retention in porous substrates [71]. Therefore, in sulfate- or chloride-rich environments, the selection of protective agents should emphasize not only the reduction in liquid–water ingress but also the maintenance of adequate vapor permeability to avoid salt accumulation and sub-surface damage.

For limestone, Manoudis et al. developed a multifunctional coating combining a silane-based precursor with ZnO NPs (0.8% *w*/*w*) for limestone protection, achieving superhydrophobicity, photocatalytic self-cleaning, and biocidal activity (94.8% inhibition for Escherichia coli and 99.9% for Staphylococcus aureus) [51]. Lisci et al. assessed silane/siloxane-based hydrophobic coatings on Portuguese limestones, showing maintained water repellency (static contact angles mostly above 90°) after thermal shock and freeze–thaw aging; however, porous Branco limestone exhibited up to ~52% flexural strength reduction due to coating-induced pore clogging, while compact Lioz and Blue limestone were more resilient [65]. Gkrava et al. also applied CaCO_3_-DA NP/KSE100 composite coatings on limestone, achieving superhydrophobicity and good mechanical, chemical, and thermal stability [67]. Armal et al. [68] included four limestones and a calcitic dolomite in case studies, with FK-3 coating effective for carbonate stones, and Dias et al. [69] found modified silanes/siloxanes derivatives most compatible on two limestones.

**Figure 7:**
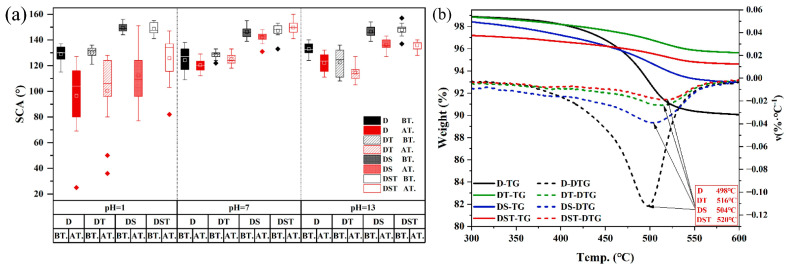
Chemical resistance and thermal stability of the nanocomposite coating system: (**a**) contact angle changes after 24 h immersion in solutions of different pH, comparing the conditions before and after treatment; and (**b**) TG (solid lines) and DTG (dotted lines) curves showing the thermal decomposition behavior, with the temperatures corresponding to the maximum mass-loss rates indicated in the figure [60].

Sandstone protection has seen significant advancements with nanocomposite coatings. Liu et al. developed an FAS-SiO_2_/FEVE-PMMA composite coating for outdoor-exposed sandstone, achieving superhydrophobicity excellent stability under acid rain, mechanical abrasion, and temperature, with ~79.8% capillary water absorption reduction and ~18.4% relative decrease in water vapor permeability, and minimal color alteration [6]. Chai et al. introduced FZS coating on sandstone, exhibiting amphiphobicity (WCA > 160°, OCA > 140°) and outstanding UV resistance [52]. Peng et al. prepared a DTMS-based nanocomposite coating with SiO_2_ and TiO_2_ NPs on sandstone, yielding superhydrophobicity (WCA > 152°, roll-off angle <10°) (Figure 7a), enhanced thermal stability (Figure 7b), UV shielding (>95%), and preserved breathability (82% water vapor permeability) [60]. Shu et al. found TSCM-treated sandstone sustained four times more salt immersion–drying–freezing cycles than organic polymer coatings before deterioration [63].

In terms of granite, Armal et al. included one granitoid in their ten natural stone case studies, selected for diverse physical, chemical, and mineralogical properties to comprehensively compare hydrophobic coatings, though specific performance details for granitoid were not elaborated beyond contributing to coating effectiveness assessment across stone types [68].

Besides physicochemical and durability-related considerations, economic criteria should also be taken into account when selecting protective materials for stone surfaces [72]. For large-scale protection, cost-effectiveness, ease of application, curing efficiency, maintenance interval, and adaptability to variable outdoor conditions are often decisive factors, which may favor relatively simple and scalable silane/siloxane or water-based systems. By contrast, for individual users or localized heritage interventions, higher-cost materials may still be acceptable when they offer advantages such as better esthetic compatibility, reversibility, self-cleaning ability, or substrate-specific performance. Therefore, the optimal choice of organic or inorganic protective agents is not identical for large-area engineering applications and for small-scale or individualized treatments.

### 4.2. Comprehensive Evaluation of Protective Effects

The comprehensive evaluation of protective coatings involves assessing their efficacy in mitigating key deterioration mechanisms, including water ingress, biological colonization, graffiti, and pollutant deposition. For water ingress mitigation, hydrophobic coatings play a crucial role. In 2018, Amir Ershad-Langroudi et al. demonstrated that acrylic, silane, and organic–inorganic nanocomposite coatings can effectively provide hydrophobic surfaces, reducing water penetration into stone pore structures [39]. Among these, silane/siloxane-based nanocomposite coatings show durable hydrophobic protective effects while maintaining water vapor permeability, preventing liquid water ingress without hindering moisture evaporation. Similarly, in 2013, Chrysi Kapridaki et al. reported that their designed transparent hydrophobic TiO_2_·SiO_2_-PDMS nanocomposite coating on marble surfaces significantly decreased the water capillary absorption coefficient [50]. For porous stones like travertine, in 2022, Mehdi Torabi-Kaveh et al. found that resin–anatase TiO_2_ nanoparticle hybrid coatings exhibited improved resistance to deterioration from salt crystallization and thermal shock, with the lowest mass changes and highest surface hardness after accelerated aging tests [49]. Additionally, in 2024, Li et al. developed a flexible–rigid hybrid siloxane oligomer that penetrates weathered stone to form a three-dimensional consolidation network, effectively strengthening the structure and resisting water ingress, as evidenced by enhanced mechanical properties [64]. Superamphiphobic coatings have also shown promise, with in 2024, Panagiotis N. Manoudis et al. [56] producing coatings with WCA and OCA > 150° by blending silane with a water-based fluoropolymer and silica NPs, exhibiting excellent water repellency.

In terms of biological colonization, several coatings have demonstrated antimicrobial properties. Chrysi Kapridaki et al. [50] further showed that their TiO_2_·SiO_2_-PDMS coating exhibited photocatalytic self-cleaning properties by removing biofilm from treated marble specimens under UV irradiation. Liliana Marinescu et al. [73] developed a two-step surface modification method involving siloxane coupling agents and silver NPs (AgNPs), which achieved significant inhibition of microbial biofilms (*Bacillus subtilis*) with reductions in biofilm formation of approximately three to four logarithmic units compared to untreated controls.

For graffiti mitigation, anti-graffiti coatings function by generating low surface energies to limit graffiti adhesion. Vera Gomes et al. distinguished between temporary coatings based on waxes and silicones with limited durability and permanent coatings such as polyurethanes, fluorocarbons, and alkyl alkoxy silanes [74]. Among tested commercial anti-graffiti coatings, polyurethane-based coatings achieved the highest reduction in capillary water absorption and minimal color and gloss changes, while wax-based coatings had lower effectiveness and sometimes altered color. The porosity of stone substrates was identified as a main factor influencing both graffiti penetration and cleaning performance of the coatings.

Regarding pollutant deposition, photocatalytic and self-cleaning properties are key. Chrysi Kapridaki et al. [50] demonstrated the degradation of organic pollutants (e.g., methylene blue stains) by their TiO_2_·SiO_2_-PDMS coating. The superamphiphobic coatings developed by Panagiotis N. Manoudis et al. [56] also showed potential in resisting oil-based pollutants due to their high oleophobicity, with the combined effect of fluoropolymer and silica NPs contributing to this performance.

### 4.3. Key Factors Influencing Protection Performance

The protection performance of stone surface coatings is influenced by a complex interplay of critical factors, including stone mineralogy, coating composition, environmental conditions, and application protocols.

Stone mineralogy and intrinsic properties are foundational to protective outcomes. The type, amount, and rate of stone decay depend largely on intrinsic properties such as mineralogical composition, texture, structure, porosity, and mechanical properties [5]. For instance, granite’s mineralogical features, including grain size and polymineral character, strongly influence cleaning effectiveness and coating interaction, with coarse-grained granites yielding higher graffiti cleaning rates than fine-grained ones [66]. Porosity variations between stone types, such as the highly porous Lecce stone (42% open porosity) versus the compact Trani stone (2% porosity), directly affect treatment efficacy and coating penetration [75]. Additionally, the interaction between stone mineralogy and coating composition is crucial for compatibility and durability, as seen in SiON coatings forming chemical bonds with hydroxyl groups in sandstone. The mineralogical properties and microstructural characteristics of stones further affect their susceptibility to biodeterioration, necessitating tailored protective materials. Substrate roughness and mineral composition also influence coating wettability, a factor often overlooked when using smooth substrates like glass slides instead of natural stones.

The initial damage state of the substrate, especially whether the stone surface is cracked or uncracked, is an important factor in selecting a protective agent. Although many published studies focus on stone type, porosity, and environmental exposure rather than directly comparing cracked and intact substrates, the available evidence shows that cracks, pores, and other discontinuities strongly affect water ingress, stress development, and subsequent deterioration [76]. In cracked or structurally weakened stones, protective materials should not be chosen only for their surface hydrophobicity; instead, systems with suitable penetration, consolidation capacity, compatibility, and strong adhesion are more appropriate because they can enter fissures, enhance cohesion, and help limit further crack propagation. By contrast, for uncracked and structurally sound surfaces, the main role of protection is often to reduce liquid–water ingress, staining, or biological fouling while maintaining water vapor permeability and visual compatibility [77]. Therefore, the cracked/uncracked condition should be regarded as a primary decision criterion in the selection of stone protective agents.

**Figure 8:**
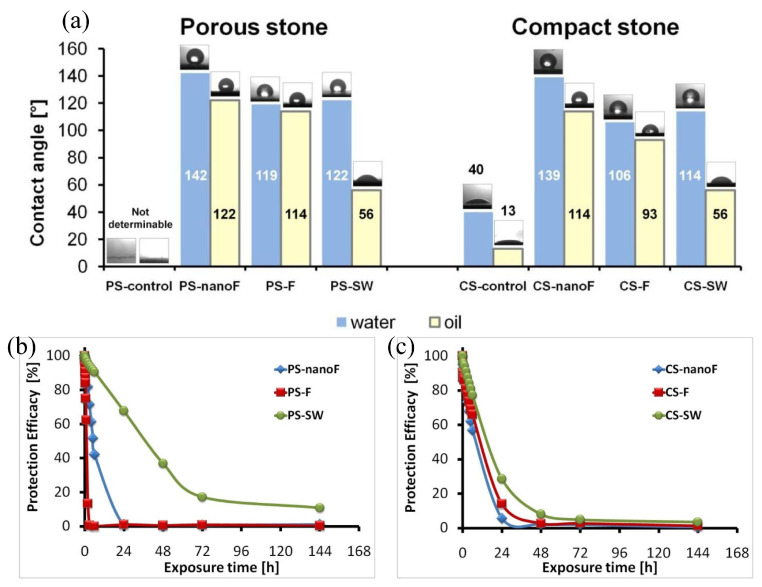
Wetting characteristics and capillary protection performance of the coating system: (**a**) WCA and OCA with representative droplets on the treated stone surfaces; time-dependent protection efficiency against capillary water absorption for (**b**) porous stone and (**c**) compact stone [75].

Coating composition significantly impacts protective performance, with formulations incorporating NPs and functional groups enhancing key properties. Inorganic NPs added to polymer coatings improve consolidation, hydrophobicity, mechanical strength, and resistance to weathering and microbial growth. For example, a fluorine resin coating containing 10 wt% SiO_2_ NPs (nanoF) demonstrated superior hydrophobicity and oleophobicity (WCA ~140°, OCA ~120°) (Figure 8a) compared to commercial fluorine-based and siloxane-based (SW) products (Figure 8b,c) [75]. SiON coatings derived from perhydropolysilazane (PHPS) showed stronger adhesion and denser protective layers than acrylic resin B72 or PDMS on sandstone, attributed to chemical bonding with the substrate. Nanoparticle concentration is critical: WCA increases with nanoparticle concentration, reaching a superhydrophobic plateau (WCA > 150°) at concentrations >0.5% *w/v* [36]. Specific polymeric composites, such as polyacrylate/silica hybrids and TiO_2_ NPs/fluoropolymer composites, exhibit quantified improvements in water uptake reduction, contact angles, and colorimetric stability [19].

Environmental conditions play a vital role in coating durability and effectiveness. External factors including weather, atmospheric composition, living agents (plants, fungi, bacteria), UV exposure, humidity, temperature variations, and pollution influence coating aging and stability. For example, biological colonization preferentially develops in rural, low-pollution areas, while black crusts form in polluted urban environments linked to sulfur dioxide. Simulated environmental stressors, such as pancreatin application to mimic bird excreta, revealed that nanoF-treated stones exhibited minimal color changes and preserved permeability, indicating enhanced resistance to biological degradation. Salt weathering cycles demonstrated that SiON coatings resist salt crystallization damage better than organic coatings due to smaller sodium sulfate crystal sizes and lower crystallization pressure. Climatic exposure can degrade nanoparticle coatings, as shown by a slight reduction in nano-TiO_2_ surface coverage (from ~91.8% to ~88.2%) under extreme simulated conditions [78]. Freeze–thaw cycles also contribute to stone decay by inducing mechanical stresses, necessitating coatings that balance liquid water reduction and water vapor permeability.

Application protocols, including coating dosage, method, and curing conditions, directly affect protective outcomes. Coating penetration depth is critical; insufficient penetration can cause mechanical stresses leading to detachment, while increasing application time or repeating applications improves penetration. For porous stones, higher product amounts are required (e.g., 150 g/m^2^ for nanoF on Lecce stone vs. 50 g/m^2^ on Trani stone), with brush application followed by curing at controlled temperature and humidity [75]. Application methods such as dip coating and curing parameters (e.g., 70 °C for varied times for PHPS) influence coating uniformity and microstructure, with SiON and B72 forming more uniform coatings than PDMS [79]. Operator skill and controlled parameters (e.g., laser wavelength, fluence for cleaning) also impact outcomes. Nanoparticle dispersion and concentration are key: uniform distribution and adequate concentration enhance mechanical strength and consolidation. For large-scale applications, methods must be suitable for atmospheric conditions, use cost-effective materials, and accommodate diverse stone types. Application methods (brushing, dip coating, spraying) and solvent systems further influence coating uniformity and penetration depth, with quantified differences in water uptake reduction and contact angles based on stone type and coating composition.

## 5. Research Gaps and Future Directions

Overall, the reviewed studies indicate that there is no universal protective coating suitable for all stone substrates and service conditions. Instead, different composite systems are more appropriate for different lithologies, deterioration mechanisms, and protection objectives. Among the currently reported materials, silane/siloxane-based hybrid systems modified with SiO_2_, TiO_2_, ZnO, or fluorinated components appear to be among the most versatile solutions for improving water repellency while maintaining relatively good durability on marble, limestone, and sandstone. In particular, TiO_2_–SiO_2_–PDMS and ZnO-containing siloxane composites are promising for applications requiring photocatalytic self-cleaning and antimicrobial performance, whereas hydroxyapatite- and calcium oxalate-related systems exhibit greater chemical affinity and consolidation potential for carbonate stones, making them especially relevant in compatibility-sensitive conservation scenarios. In addition, fluorinated ZnO/SiO_2_ systems, FAS-SiO_2_/resin composites, and resin–TiO_2_ hybrid coatings have demonstrated excellent hydrophobic or amphiphobic behavior as well as improved resistance to UV radiation, salt weathering, or chemical attack.

At the same time, the available evidence also shows that the advantages of these systems are often substrate-dependent and may be accompanied by important limitations. For highly porous stones, some hydrophobic treatments may reduce water ingress effectively but also increase the risk of pore blocking, moisture retention, or mechanical incompatibility under freeze–thaw conditions. Superhydrophobic and superamphiphobic systems, although highly effective in wettability control, still face challenges related to long-term durability, reproducibility, and resistance to acid rain, abrasion, and outdoor aging. Therefore, future research should focus not only on developing water-based and low-VOC formulations, but also on establishing substrate-oriented material selection criteria, balancing hydrophobicity with vapor permeability and compatibility, and performing more systematic long-term evaluations under realistic service environments. Advanced non-destructive characterization methods, such as X-ray CT and micro-CT [80], should also be further introduced to reveal pore-structure evolution, crack connectivity, and the penetration/distribution behavior of protective agents inside stone substrates, thereby improving the mechanistic understanding and practical evaluation of stone protection systems.

## Data Availability

The original contributions presented in this study are included in the article. Further inquiries can be directed to the corresponding authors.
